# Integrated pharmacokinetic–Pharmacodynamic (PK/PD) model to evaluate the in vivo antimicrobial activity of Marbofloxacin against *Pasteurella multocida* in piglets

**DOI:** 10.1186/s12917-017-1099-z

**Published:** 2017-06-15

**Authors:** Qing Lin Zeng, Xian Mei, Jia Su, Xiao Hong Li, Wen Guang Xiong, Yan Lu, Zhen Ling Zeng

**Affiliations:** 1 National Laboratory of Safety Evaluation of veterinary Drugs, College of Veterinary Medicine, South China Agricultural University, Guangzhou, China; 2 National Laboratory of Safety Evaluation (Environmental Assessment) of Veterinary Drugs, College of Veterinary Medicine, South China Agricultural University, Guangzhou, China

**Keywords:** Marbofloxacin, PK/PD, *P. Multocida*, Tissue-cage model, Piglets

## Abstract

**Background:**

Marbofloxacin is a veterinary fluoroquinolone with high activity against *Pasteurella multocida.* We evaluated it’s in vivo activity against *P. multocida* based on in vivo time-kill data in swine using a tissue-cage model. A series of dosages ranging from 0.15 to 2.5 mg/kg were administered intramuscularly after challenge with *P. multocida* type B, serotype 2.

**Results:**

The ratio of the 24 h area under the concentration-time curve divided by the minimum inhibitory concentration (AUC_24_TCF/MIC) was the best PK/PD index correlated with the in vivo antibacterial effectiveness of marbofloxacin (*R*2 = 0.9279). The AUC_24_TCF/MIC necessary to achieve a 1-log_10_ CFU/ml reduction and a 3-log_10_ CFU/ml (90% of the maximum response) reduction as calculated by an inhibitory sigmoid E_max_ model were 13.48 h and 57.70 h, respectively.

**Conclusions:**

Marbofloxacin is adequate for the treatment of swine infected with P. *multocida.* The tissue-cage model played a significant role in achieving these PK/PD results.

## Background

The increase in drug-resistance coupled with the lack of new and effective antimicrobials indicates that modified drug-dosage regimens may be useful clinical alternatives. In the clinic, pharmacokinetic (PK) and pharmacodynamic (PD) outcomes are interrelated [1]. PK/PD modeling in veterinary medicine can be used to define the relationship between the effects of different drug concentrations and therapeutic outcomes. In short, PK/PD modeling provides a chance for dosage optimization [2]. We used the tissue cage (TC) infection model to evaluate PK and PD profiles in interstitial fluids in the presence of an active immune response [3–5].

*P. multocida* is a widespread pathogen that inhabits mucosal surfaces and upper respiratory tracts of clinically healthy animals [6]. It is the causative agent of fowl cholera in poultry, atrophic rhinitis in swine and hemorrhagic septicemia in buffalo and cattle [7–10]. It also plays a major role in pneumonia in swine and ruminants as an opportunistic pathogen [11].

Marbofloxacin is a synthetic third-generation fluoroquinolone that is used solely in veterinary medicine [12, 13]. It is active against Gram-negative and some Gram-positive bacteria as well as mycoplasmas and other intracellular pathogens [14, 15]. It is widely used against most of the swine respiratory pathogens including *Actinobacillus pleuropneumoniae*, *Haemophilus parasuis and P. multocida* [16–19].

Marbofloxacin acts as a bactericidal concentration-dependent antibiotic similar to other fluoroquinolones. The AUC_24_/MIC or C_max_/MIC (peak concentration divided by the MIC) are the PK/PD surrogates that most closely reflect clinical outcomes*.* [20, 21]. We used an integrated PK/PD model to evaluate the in vivo antimicrobial activity of marbofloxacin against *P*. *multocida* in swine. This approach gives an approximation of the most effective treatment for achieving a bacteriological cure and minimizing the emergence of bacterial resistance.

## Methods

### Animals and tissue-cages

Twenty clinically healthy castrated crossbred piglets (~1-month Duroc ×Landrace × Yorkshire),purchased from Guangdong pig breeding farm, with body weights of 14 to 18 kg were included in the study. These animals were housed in individual pens for a week prior to experiments with a controlled temperature of 26 °C. The animals were fed antimicrobial-free feed twice a day with water available ad libitum. Tissue-cages were fabricated according to a previously published method [5]. Two tissue-cages were implanted subcutaneously in the neck of each piglet after sedation and local anesthesia. About 3–4 weeks later and after wound healing, 0.5 mL tissue-cage fluid (TCF) was sampled by percutaneous puncture. TCF samples were cultured for aerobic and anaerobic contaminants to ensure sterility before commencing the experiments.

### Bacterial strain and inoculant preparation

*P. multocida* strain CVCC434 (type B, serotype 2) was purchased from the China Veterinary Culture Collection Centre (Beijing, China). The original bacterial culture was isolated from a piglet that died of plague in Jiang Su, China. *P. multocida* was grown on tryptic soy agar (Guangdong Huaikai Microbial, Guangzhou, China) supplemented with 5% defibrinated sheep blood (BTSA). Bacteria were cultured in Mueller–Hinton broth (Becton Dickinson, Sparks, MD, USA), with shaking at 37 °C and grown to 10^9^ CFU/mL. The cells were then concentrated by centrifugation at 3000×g for 10 min and suspended in sterile 0.9% NaCl to 10^8^ CFU/mL.

### Drugs

Marbofloxacin was obtained as a 10% injectable aqueous solution from Yuan Zhen (Hebei, China). Marbofloxacin standard was purchased from Dr. Ehrenstorfer (GmbH) and ofloxacin was acquired from the National Institute for Food and Drug Control (Beijing, China). Pentobarbital sodium was from Jian Yang Biotechnology and procainamide hydrochloride was purchased from Xin Zheng (Tianjin).

### MIC determinations

MIC values were determined by an agar dilution method as a preliminary screening according to Clinical and Laboratory Standards Institute (CLSI) reference methods. In this study, we measured the MIC of marbofloxacin against the *P. multocida* in the tissue-cage fluid by a micro dilution assay in triplicate using the tissue-cage fluid as matrix.

### PK studies

Tissue cage fluid (0.5 ml) was sampled 1 h after the injection of drug and thereafter at 3, 6, 9, 12, 24, 30, 36, 48, 72 and 96 h. All the samples were centrifuged at 6000×g at 4 °C for 10 min. The supernatants were stored at −20 °C. The concentrations of marbofloxacin in tissue cage fluid were determined using a Waters 2695 series high performance liquid chromatography (HPLC) system with fluorescence detection [13, 22]. The chromatographic separation was achieved on an Agilent BDS C18 column (250 mm × 4.6 mm; internal diameter, 5 μm) at 28 °C with a thermostat column oven (Agilent 1200 series). All TCF samples were thawed at room temperature before analysis and 3 mL of trichloromethane was added to a 10 mL centrifuge tube with 200 μL tissue-cage fluids spiked with 10 μL ofloxacin internal standard, and vortexed for 1 min. The aqueous layer was discarded after centrifugation at 6000 g for 10 min and the organic layer was dried under a stream of nitrogen. The residue was suspended with 0.2 mL mobile phase and a 10 μL aliquot was taken and injected into HPLC for analysis after filtered through a 0.22 μm nylon syringe filter (JinTeng Experiment Equipment, Tianjing). The determination of marbofloxacin in the tissue cage fluid was linear within a range of 0.01–2 μg/mL and the correlation coefficient was >0.99. The lower limit of quantitation was 0.01 μg/mL. The recoveries of marbofloxacin in tissue-cage fluid were >85%. The standard deviations (SD) and the coefficients of variability (CV %) for both interday and intraday were *<*8% in TCF.

### Tissue-cage infection and in vivo kill curve of marbofloxacin

Twenty piglets were divided into ten groups randomly, with 4 tissue-cages for each group. 24 h after 0.5 mL of *P. multocida* saline suspension (~2.0 × 10^8^ CFU/mL) were injected into the sterile tissue cage; nine groups were treated with a series of marbofloxacin intramuscular injections (10%) at 0.15, 0.3, 0.5, 0.8, 1.0, 1.3, 1.6, 2.0, 2.5 mg/kg. The control group was given sterile physiological saline only. TCF samples were aspirated from the tissue-cages by percutaneous puncture at 0, 3, 6, 9, 12 and 24 h after dosing. 100 μL of tissue cage fluid was serially diluted 10-fold in saline within 1 h of sampling for CFU determinations. From each dilution, 20 μL was spread on BTSA plated in triplicate and incubated for 16 h at 37 °C.The limit of detection was 500 CFU/mL.

### Protein binding

To determination the protein binding of marbofloxacin in TCF, marbofloxacin at 0.05, 0.1, 0.5, 1 and 2 mg/L was spiked into pooled uninfected TCF in triplicate. An Amicon Centrifree Micropartition device with a 10 KDa cutoff (Millipore, Bedford, MA, USA) was used to remove the protein-bound drug as previously described [23, 24]. The marbofloxacin concentration before and after ultrafiltration and centrifugation was determined as described in the PK studies. The percent of drug bound to protein was calculated as the following equation: Bound % = (1-Cu/Ci) × 100%, where Cu is the filtrate concentration and Ci is the initial concentration.

### Pharmacokinetic-Pharmacodynamic analysis

The MIC value was included in the calculation of the PK/PD index. According to standard methodology, an inhibitory sigmoid E_max_ model was used to assess the PK/PD index with the highest predictive value to the different indices for each dosing regimen (Eq. 1). The R^2^ value was computed for the correlation between antimicrobial effectiveness and each of the PK/PD parameters.

1 $$ E={E}_{max}-\left({E}_{max}-{E}_0\right)\times {C}_e^N/\left({E C}_{50}^N+{C}_e^N\right) $$

*E* is the antibacterial effect; E_0_ represents maximum drug effect 24 h after *i.m* administration. *E*_*max*_ is the change of bacterial load (Log_10_ CFU/mL) in the infected tissue-cage with no drug present. *C*_*e*_ is the PK/PD index magnitude. *EC*_*50*_ is the magnitude required for achieve 50% of *E*_*max*_ and *N* is the Hill coefficient that describes the sigmoid shape. PK and PD indices were calculated using a non-compartment model in WinNonlin 6.2 software (Pharsight Corporation, Mountain View, CA, USA),

## Results

### MIC of marbofloxacin for *P. multocida*

Tissue cage fluid was sterile as assessed by aerobic and anaerobic culture. We then determined that the MIC of marbofloxacin for *P. multocida* in tissue cage fluid was 0.12 μg/mL ex vivo. This did not change after exposure to marbofloxacin for 24 h and this number was used for PK/PD calculations.

### TC infection and in vivo kill curve of marbofloxacin

**Fig. 1:**
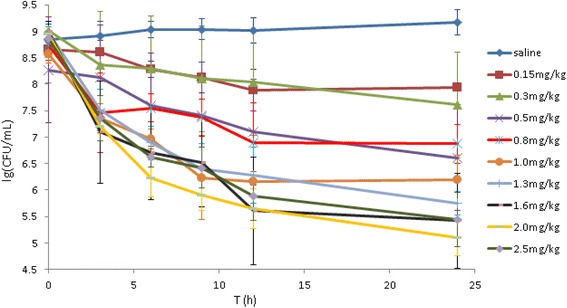
In vivo time killing curve of marbofloxacin against *P. multocida* in TCF. Kb-1 means the group treated by saline. Mean log_10_CFU/mL values only (*n* = 4 experimentations). (Excel, Microsoft Office)

We determined the in vivo antibacterial activity of marbofloxacin against P. *multocida* CVCC434. *P. multocida* inoculated into tissue cages in the absence of antibiotic was 8.79 ± 0.22 log_10_ CFU/mL averaged over all tissue cages. When marbofloxacin was administered intramuscularly, the bacterial counts for marbofloxacin-treated groups decreased by 0.72 to 3.85log_10_ CFU/mL. By this time, the control saline-treated cages had increased to 9.17log_10_ CFU/mL. Marbofloxacin exerted bactericidal activity when the dosage via intramuscular injection exceeded 1.3 mg/kg (Fig. 1).

### PK study

**Fig. 2:**
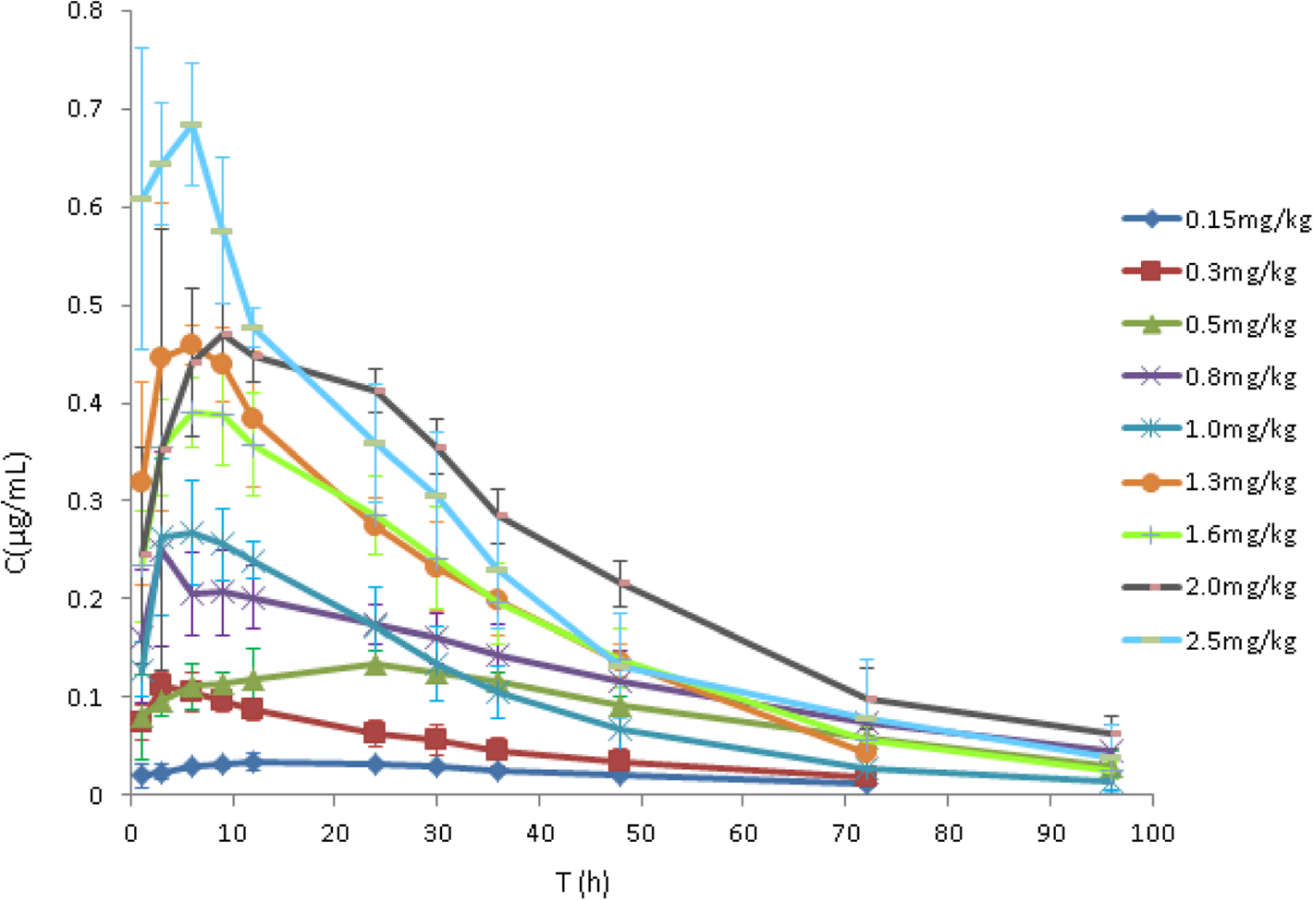
Pharmacokinetic profiles of marbofloxacin in TCF. The tissue-cages were infected with *P. multocida* as described above, 24 h later administered with single intramuscularly doses of 0.15, 0.3,0.5,0.8,1.0,1.3,1.6,2.0 and 2.5 mg/kg marbofloxacin as BW. All TCF were sampled by puncture at 1, 3, 6, 9, 12, 24, 30, 36, 48, 72and 96 h after dosing. Marbofloxacin concentrations were determined using a HPLC method with fluorescence detection. But a small number of samples sampled on 96 h were undetected. Each symbol represents the mean ± standard deviation of the levels in the four tissue-cages. (Excel, Microsoft Office)

**Fig. 3:**
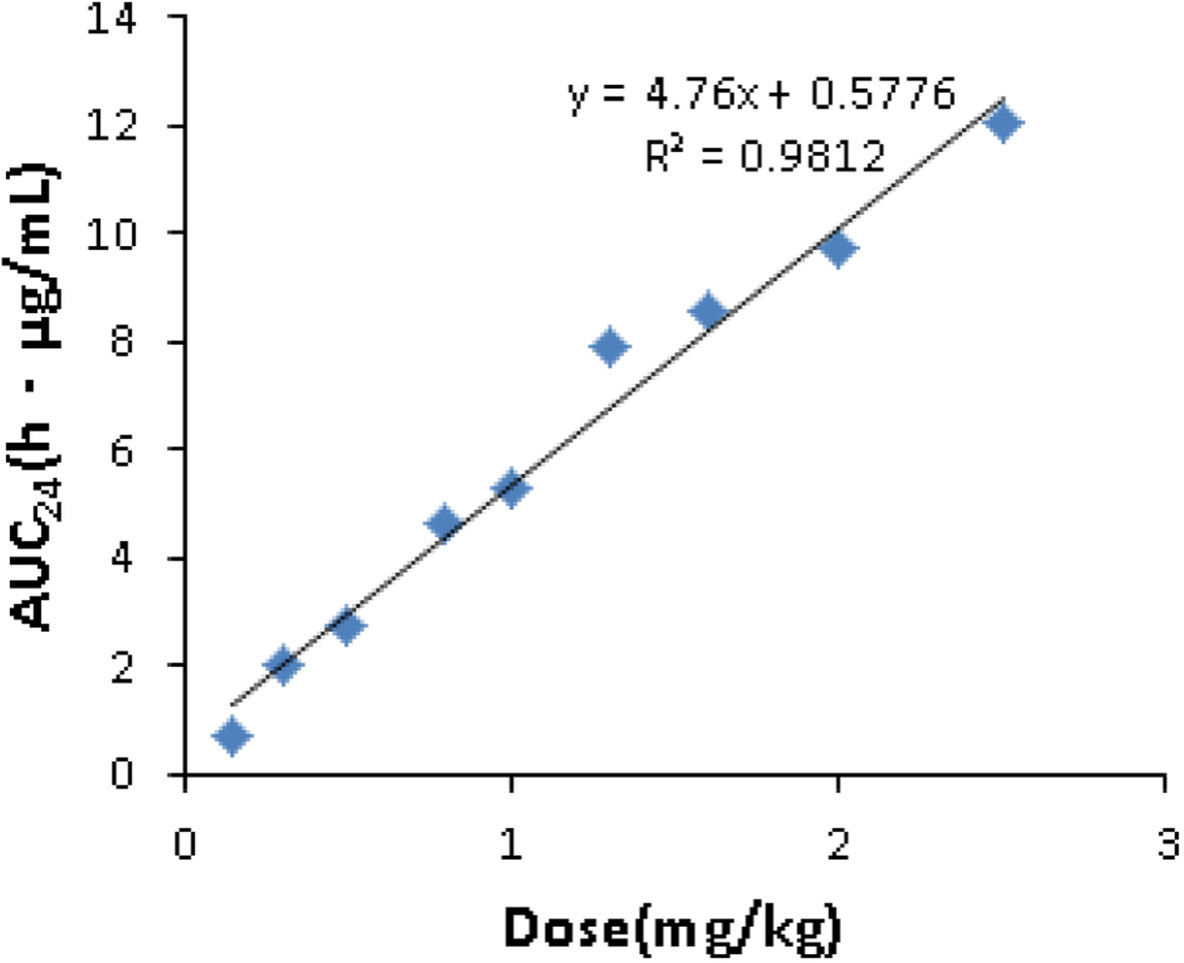
Linear regression plots between administered doses and AUC_24_ values. (Excel, Microsoft Office)

The concentration-time profile of marbofloxacin in the infected cages were calculated as AUC_24h_ and C_max_ of marbofloxacin in the tissue cage fluid following multiple dosages obtained from the measured marbofloxacin concentration data from each cage (Fig. 2 and Table 1). The mean values of AUC_24_ for marbofloxacin increased with dose escalation in the tissue cage fluid. A significant correlation (*R*_2_ = 0.9812) was observed between AUC_24_ TCF and dosages (Fig. 3).

**Table 1:** AUC_24_ and Cmax of marbofloxacin following multiple dosages in the *P. multocida* infected tissue cage fluid

Doses(mg/kg)	AUC_24_(h·μg/mL) (X^a^ ± SD)	C_max_(μg/mL) (X^a^ ± SD)
0.15	0.72 ± 0.09	0.04 ± 0.01
0.3	2.04 ± 0.23	0.11 ± 0.01
0.5	2.70 ± 0.20	0.14 ± 0.01
0.8	4.65 ± 0.68	0.27 ± 0.07
1.0	5.25 ± 0.40	0.31 ± 0.07
1.3	7.89 ± 1.49	0.51 ± 0.06
1.6	8.53 ± 0.70	0.53 ± 0.06
2.0	9.70 ± 0.82	0.54 ± 0.12
2.5	12.03 ± 0.56	0.74 ± 0.10

X^a^ is the mean value.

### PB

The protein binding of marbofloxacin in tissue cage fluid was 47.89 ± 1.86% and 52.90 ± 3.30% in serum within the range of 0.05 ~ 2 mg/L for marbofloxacin (Table 2).

**Table 2:** Protein binding of marbofloxacin in serum and tissue cage fluid

Spiked concentration (μg/mL)	Protein binding (X^a^ ± SD)	
	Serum	TCF
0.05	52.17 ± 3.36	45.71±1.30
0.1	53.84 ± 2.13	48.68±1.39
0.5	48.92 ± 2.68	46.99±0.73
1	55.58 ± 0.35	48.20±1.66
2	53.98 ± 1.42	49.90±0.40
total	52.90 ± 3.30	47.89±1.86

### Magnitude of the PK/PD parameter required for efficacy

**Fig. 4:**
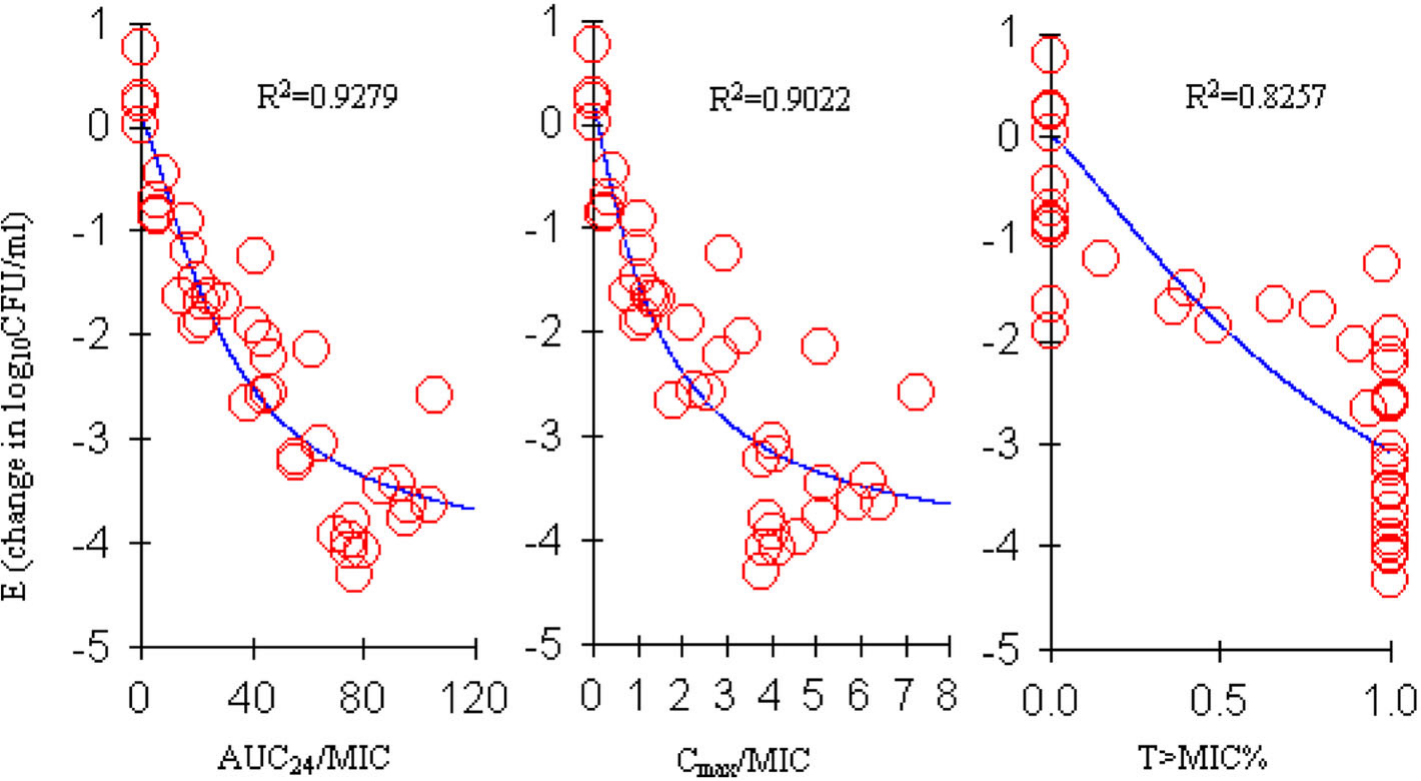
Relationships between PK/PD parameters (AUC/MIC, Cmax/MIC, T > MIC %) and antibacterial effect (E) after 24 h of therapy. *R*2 is the correlation coefficient. (Winnonlin software)

The PK parameters best predicting the in vivo of the drug were the AUC_24_/MIC (R^2^ = 0.9279), %T *>* MIC (*R* ^2^ = 0.8257) and C_max_/MIC (R^2 =^0.9022) (Fig. 4). The magnitudes of AUC_24_/MIC predicted for a 1-log reduction and a 3-log reduction (bactericidal) were 13.48 h and 57.70 h, respectively, and the slope of both parameters for antibacterial effectiveness was 1.41 (Table 3).

**Table 3:** PK/PD analysis of marbofloxacin in TCF infection model

Parameter(unit)	Value
E_max_ (log_10_(CFU/mL))	0.05
E_0_(log_10_(CFU/mL))	−4.20
AUC_24_/MIC for 1log reduction(h)	13.48
AUC_24_/MIC EC_50_(h)	29.78
AUC_24_/MIC for bactericidal action(h)	57.70
Slope(N)	1.41

## Discussion

Respiratory diseases greatly affect the production and health of swine and result in high economic losses [25]. In Europe, *P. multocida* is one of the most common bacterial species among porcine respiratory diseases and leads to atrophic rhinitis in young pigs [26]. Marbofloxacin shows excellent effectiveness for treating *P. multocida* infection through in vitro tests [16]. The protein binding of marbofloxacin was low in previous studies and was 52% for serum, 40% for tissue cage fluid in cattle and 49.4% for pig serum [3, 16]. Only free drug in the interstitial fluid is microbiologically active against extracellular bacteria [3, 27].

In the present study, the protein binding of marbofloxacin in pig serum and TCF were 52.90% and 47.89% respectively and these values were independent of concentration in the range of 0.05 ~ 2 μg/mL. The AUC_24_TCF of marbofloxacin following single intramuscular administration (2.5 mg/kg) was estimated to be 12.03 h·μg/mL, which was lower than that in plasma (25.8 h·μg/mL) [28]. The C_max_ for tissue cage fluid was lower than previously reported in plasma (2.57 μg/mL) and tissue cage fluid (1.57 μg/mL) in pigs [28, 29].

The PK/PD model integrating the PK parameter, the in vitro MIC and PD outcome are all valuable in designing rational dosage regimens and in the measurement of susceptibility breakpoints [20, 30]. Our data indicated that AUC_24_TCF/MIC was the best parameter that correlated with the in vivo antibacterial effects of marbofloxacin against *P. multocida.* This agrees with previous studies of marbofloxacin activity against *P. multocida* [3, 31]. We found an AUC_24_TCF/MIC value of 100.25 h (2.5 mg/kg) that was lower than the reported value in plasma in piglets (264.10 h) [28].

The AUC_24h_TCF/MIC ratios that produced a 1-log reduction, and 50% and 90% of the maximum effect were 13.48 h, 29.78 h and 57.70 h, respectively. The AUC_24_TCF/MIC ratio for bactericidal effects in this study was different from previous study values of 31.29, 278.08 and 50.65 h for 3-log reductions [3, 22, 31]. These differences may by accounted for by examining the ex vivo PK/PD model. This model holds the antibiotic concentration constant but this concentration would vary with time in the animal infection model [32]. In addition, the ex vivo and neutropenic murine lung infection models do not take the host immune responses into consideration. Finally, the magnitude of PK/PD indices were influenced by the bacterial load and this may influence characteristics of the PK parameters. A higher antimicrobial concentration would be required for higher pathogen loads [31, 33].

An AUC_24_/MIC ratio exceeding a value of 125 h was reported as useful for the treatment of infections caused by Gram-negative organisms while reducing the risk of resistance induction [34–36]. In combination with the MIC_90_ (0.06 μg/mL) from two previous studies, and the PK characteristics in this study, we can infer that a dosage regimen of marbofloxacin 2.0 mg/kg (AUC_24_TCF/MIC, 161.67 h > 125 h) was effective for the treatment of *P. multocida* infections [37, 38]. However, considering that we found an AUC_24_TCF/MIC ratio of 57.70 h that produced a 3-log reduction in this investigation, a marbofloxacin dose of 1.34 mg/kg every 24 h is recommended for treatment of *P. multocida* infections with MIC values lower than 0.12 μg/mL. The dosage 1.34 mg/kg was calculated by the equation *AUC*_24_ = 4.76^∗^Dose + 0.5776 (Fig. 3). However, we did not assess the diffusion of marbofloxacin in lungs and the immune responses in lungs may be different from that in tissue cage fluid.

## Conclusions

The present study characterized the in vivo effectiveness of marbofloxacin against *P. multocida* in a tissue cage model in pigs. The AUC_24_TCF/MIC proved to be the PK/PD index that predicted the antimicrobial activity of marbofloxacin against *P. multocida.* Marbofloxacin presented excellent antibacterial activity with an AUC24TCF/MIC ratio of 57.70 h for a 3-log reduction in bacteria. Although this study needs to be validated by clinical treatment under practical conditions, the results indicated that it may be a critical step closer to clinical trials for optimization of dosage regimens.

## Abbreviations

AUC: area under the curve
AUC_24h_/MIC: the ratio of area under the concentration-time curve over 24 h to MIC
BW: body weight
CFU: Colony forming unit
CLSI: clinical and laboratory standards institute
C_max_/MIC: the ratio of peak concentration to MIC
Cmax: peak concentration
CVCC: China veterinary culture collection
HPLC: high performance liquid chromatography
MIC: minimum inhibitory concentration
*P. multocida*: Pasteurella multocida;
PD: Pharmacodynamics
PK: Pharmacokinetics
T% > MIC: The percent time that marbofloxacin concentrations were above the MIC
TC: tissue cage
TCF: tissue cage fluid

## References

[1] Frimodt-Moller N (2002). How predictive is PK/PD for antibacterial agents?. Int J Antimicrob Ag.

[2] Ahmad I, Huang LL, Hao HH, Sanders P, Yuan ZH. Application of PK/PD modeling in veterinary field: dose optimization and drug resistance prediction. Biomed Res Int. 2016; 10.1155/2016/5465678 PMC4771886 26989688

[3] Cao C, Qu Y, Sun M, Qiu Z, Huang X, Huai B (2015). In vivo antimicrobial activity of marbofloxacin against Pasteurella multocida in a tissue cage model in calves. Front Microbiol.

[4] Sidhu PK, Landoni MF, Aliabadi FS, Lees P (2010). PK-PD integration and modeling of marbofloxacin in sheep. Res Vet Sci.

[5] Zhang BX, Gu XY, Li YF, Li XH, Gu MX, Zhang N, et al. In vivo evaluation of mutant selection window of cefquinome against *Escherichia coli* in piglet tissue-cage model. BMC Vet Res. 2014;10 10.1186/s12917-014-0297-1 PMC4279896 25511985

[6] Holst E, Rollof J, Larsson L, Nielsen JP (1992). Characterization and distribution of Pasteurella species recovered from infected humans. J Clin Microbiol.

[7] Blackall PJ, Pahoff JL, Marks D, Fegan N, Morrow CJ (1995). Characterisation of Pasteurella multocida isolated from fowl cholera outbreaks on turkey farms. Aust Vet J.

[8] Magyar T, King VL, Kovacs F (2002). Evaluation of vaccines for atrophic rhinitis--a comparison of three challenge models. Vaccine.

[9] Kalorey DR, Yuvaraj S, Vanjanri SS, Gunjal PS, Dhanawade NB, Barbuddhe SB (2008). PCR analysis of Pasteurella multocida isolates from an outbreak of pasteurellosis in Indian pigs. Comp Immunol Microb.

[10] Aalbaek B, Eriksen L, Rimler RB, Leifsson PS, Basse A, Christiansen T (1999). Typing of Pasteurella multocida from haemorrhagic septicaemia in Danish fallow deer (*Dama dama*). APMIS.

[11] Boyce JD, Seemann T, Adler B, Harper M (2012). Pathogenomics of Pasteurella multocida. Curr Top Microbiol Immunol.

[12] Schneider M, Thomas V, Boisrame B, Deleforge J (1996). Pharmacokinetics of marbofloxacin in dogs after oral and parenteral administration. J Vet Pharmacol Ther.

[13] Aliabadi FS, Lees P (2002). Pharmacokinetics and pharmacokinetic/pharmacodynamic integration of marbofloxacin in calf serum, exudate and transudate. J Vet Pharmacol Ther.

[14] Meunier D, Acar JF, Martel JL, Kroemer S, Valle M (2004). Seven years survey of susceptibility to marbofloxacin of bovine pathogenic strains from eight European countries. Int J Antimicrob Agents.

[15] Thomas A, Nicolas C, Dizier I, Mainil J, Linden A (2003). Antibiotic susceptibilities of recent isolates of mycoplasma bovis in Belgium. Vet Rec.

[16] Dorey L, Hobson S, Lees P (2016). Potency of marbofloxacin for pig pneumonia pathogens Actinobacillus pleuropneumoniae and Pasteurella multocida: comparison of growth media. Res Vet Sci.

[17] Appelbaum PC, Hunter PA (2000). The fluoroquinolone antibacterials: past, present and future perspectives. Int J Antimicrob Agents.

[18] Spreng M, Deleforge J, Thomas V, Boisrame B, Drugeon H (1995). Antibacterial activity of marbofloxacin. A new fluoroquinolone for veterinary use against canine and feline isolates. J Vet Pharmacol Ther.

[19] Vilalta C, Giboin H, Schneider M, El Garch F, Fraile L (2014). Pharmacokinetic/pharmacodynamic evaluation of marbofloxacin in the treatment of Haemophilus parasuis and Actinobacillus pleuropneumoniae infections in nursery and fattener pigs using Monte Carlo simulations. J Vet Pharmacol Ther.

[20] Craig WA (1998). Pharmacokinetic/pharmacodynamic parameters: rationale for antibacterial dosing of mice and men. Clin Infect Dis.

[21] MacGowan A, Bowker K (2002). Developments in PK/PD: optimising efficacy and prevention of resistance. A critical review of PK/PD in in vitro models. Int J Antimicrob Agents.

[22] Shan Q, Wang J, Yang F, Ding H, Liang C, Lv Z (2014). Pharmacokinetic/pharmacodynamic relationship of marbofloxacin against Pasteurella multocida in a tissue-cage model in yellow cattle. J Vet Pharmacol Ther.

[23] Zeitlinger M, Sauermann R, Fille M, Hausdorfer J, Leitner I, Muller M (2008). Plasma protein binding of fluoroquinolones affects antimicrobial activity. J Antimicrob Chemoth.

[24] Greko C, Finn M, Franklin A, Bengtsson B (2003). Pharmacokinetic/pharmacodynamic relationship of danofloxacin against Mannheimia haemolytica in a tissue-cage model in calves. J Antimicrob Chemoth.

[25] Dayao DA, Gibson JS, Blackall PJ, Turni C (2014). Antimicrobial resistance in bacteria associated with porcine respiratory disease in Australia. Vet Microbiol.

[26] Pors SE, Hansen MS, Bisgaard M, Jensen HE (2011). Occurrence and associated lesions of Pasteurella multocida in porcine bronchopneumonia. Vet Microbiol.

[27] Zeitlinger M, Sauermann R, Fille M, Hausdorfer J, Leitner I, Muller M (2008). Plasma protein binding of fluoroquinolones affects antimicrobial activity. J Antimicrob Chemother.

[28] Hossain MA, Hc P, Jeong K, Yh J, Kim DG, Kang J (2017). Pharmacokinetic and Pharmacodynamic Evaluation of Marbofloxacin in Pig against Korean Local Isolates of Actinobacillus pleuropneumoniae. Biomed Res Int.

[29] Ding H, Li Y, Chen Z, Rizwan-ul-Haq M, Zeng Z (2010). Plasma and tissue cage fluid pharmacokinetics of marbofloxacin after intravenous, intramuscular, and oral single-dose application in pigs. J Vet Pharmacol Ther.

[30] Andes D, Craig WA (1998). In vivo activities of amoxicillin and amoxicillin-clavulanate against Streptococcus Pneumoniae: application to breakpoint determinations. Antimicrob Agents Chemother.

[31] Qu Y, Qiu Z, Cao C, Lu Y, Sun M, Liang C (2015). Pharmacokinetics/pharmacodynamics of marbofloxacin in a Pasteurella multocida serious murine lung infection model. BMC Vet Res.

[32] Xiao X, Sun J, Yang T, Fang X, Wu D, Xiong YQ (2015). In vivo pharmacokinetic/pharmacodynamic profiles of valnemulin in an experimental intratracheal mycoplasma gallisepticum infection model. Antimicrob Agents Chemother.

[33] Illambas J, Potter T, Cheng Z, Rycroft A, Fishwick J, Lees P (2013). Pharmacodynamics of marbofloxacin for calf pneumonia pathogens. Res Vet Sci.

[34] Hyatt JM, Nix DE, Schentag JJ (1994). Pharmacokinetic and pharmacodynamic activities of ciprofloxacin against strains of Streptococcus Pneumoniae, *Staphylococcus aureus*, and Pseudomonas Aeruginosa for which MICs are similar. Antimicrob Agents Chemother.

[35] Gunderson BW, Ross GH, Ibrahim KH, Rotschafer JC (2001). What do we really know about antibiotic pharmacodynamics?. Pharmacotherapy.

[36] Thomas JK, Forrest A, Bhavnani SM, Hyatt JM, Cheng A, Ballow CH (1998). Pharmacodynamic evaluation of factors associated with the development of bacterial resistance in acutely ill patients during therapy. Antimicrob Agents Chemother.

[37] El Garch F, de Jong A, Simjee S, Moyaert H, Klein U, Ludwig C (2016). **Monitoring of antimicrobial susceptibility of respiratory tract pathogens isolated from diseased cattle and pigs across Europe,** 2009-2012. VetPath results. Vet Microbiol.

[38] de Jong A, Thomas V, Simjee S, Moyaert H, El Garch F, Maher K (2014). Antimicrobial susceptibility monitoring of respiratory tract pathogens isolated from diseased cattle and pigs across Europe: the VetPath study. Vet Microbiol.

